# The Use of Optical Genome Mapping for the Detection of Tyrosine Kinase Gene Fusions in Myeloid/Lymphoid Neoplasms

**DOI:** 10.1111/jcmm.70640

**Published:** 2025-06-18

**Authors:** Justine Vanhevel, Katrina Rack, Geneviève Ameye, Hayat Mokrani, Jolien De Bie, Lucienne Michaux, Barbara Dewaele

**Affiliations:** ^1^ Laboratory for the Cytogenetic and Molecular Diagnosis of Hematological Malignancies Centre for Human Genetics, University Hospitals Leuven Belgium; ^2^ Sorbonne Université, INSERM, Centre de Recherche Saint‐Antoine, CRSA, AP‐HP Hôpital Saint‐Antoine Paris France

**Keywords:** myeloid and lymphoid neoplasms, optical genome mapping, tyrosine kinase rearrangements

## Abstract

Myeloid/Lymphoid Neoplasms with eosinophilia and involvement of Tyrosine Kinase gene fusions (MLN‐TK) is a WHO disease category including a diverse group of malignancies characterised by recurrent genomic rearrangements of tyrosine kinase (TK) genes such as *PDGFRA*, *PDGFRB*, *FGFR1*, *JAK2*, *ETV6* and *FLT3*. Identification of these TK rearrangements is important for the accurate diagnosis of MLN‐TK and allows targeted therapy with TK inhibitors. In this study, we validated the use of optical genome mapping (OGM) retrospectively by analysing 11 samples from 10 cases with suspected or known TK rearrangements, previously analysed by current standard of care (SOC) methodologies, i.e., chromosome banding analysis (CBA), FISH and/or PCR‐based techniques. In six abnormal cases, OGM was able to detect the rearrangements previously determined by SOC methods. Furthermore, OGM identified the fusion partner in the *JAK2*‐ and *PDGFRB*‐rearranged cases and elucidated the mechanism underlying the *BCR::FGFR1* and *ETV6::SYK* rearrangement. In two cases with a normal karyotype, OGM detected two cryptic *FIP1L1::PDGFRA* and *TNIP1::PDGFRB* rearrangements. In the two remaining cases, no abnormalities were detected either by OGM or SOC methods. We demonstrate that OGM is a valid technique for the diagnostic workflow of MLN‐TK, able to detect TK rearrangements and to identify unknown TK fusion partners.

## 1 Introduction

Myeloid and Lymphoid Neoplasms with eosinophilia and Tyrosine Kinase gene fusions (MLN‐TK) is a World Health Organisation (WHO) and International Consensus Classification (ICC) disease category of rare haematological malignancies characterised by genomic rearrangements in genes encoding specific tyrosine kinases (TK). Fusion products result in constitutive activation of the kinase domain, which promotes cell proliferation and survival [1].

MLN‐TK can manifest as an extensive range of histological diagnoses, most frequently as chronic myeloid neoplasms such as myelodysplastic (MDS) and/or myeloproliferative (MPN) neoplasms and mixed entities, but they can also manifest as acute myeloid leukaemia (AML), mixed‐phenotype acute leukaemias (MPAL) and B‐lymphoblastic (B‐ALL) or T‐lymphoblastic leukaemia/lymphoma (T‐ALL/T‐LBL) [2]. Peripheral blood and bone marrow eosinophilia is common in these conditions but is not always present [3]. In adults, eosinophilia and hypereosinophilia are defined as more than 0.5 × 10^9^/L and 1.5 × 10^9^/L eosinophils in peripheral blood, respectively [4, 5].

Several TK rearrangements involving *PDGFRA, PDGFRB, FGFR1, JAK2, FLT3* and *ABL1* have been described in MLN‐TK involving a large number of different fusion partner genes (> 70) [6].

Within the group of MLN‐TK, rearrangements involving *PDGFRA* are the most frequent. In most cases, a cryptic interstitial deletion on chromosome 4q12 results in a *FIP1L1::PDGFRA* fusion gene, although other fusion partners have also been described [1, 2, 3]. Rearrangements involving *PDGFRB* are the second most common abnormality. Although *ETV6::PDGFRB* fusions [t(5;12)(q32;p13.2)] are the most frequent, more than 30 different partners have been identified [1]. Both *PDGFRA*‐ and *PDGFRB*‐rearranged neoplasms show a good therapeutic response to specific TK inhibitors (TKI) such as Imatinib [2, 3].

MLN‐TK with *FGFR1* rearrangements is a clinically diverse group characterised cytogenetically by a t(8;13)(p11.2;q12) or variant translocation. Currently, 14 partner genes have been identified. Unlike MLN‐TK with *PDGFRA* or *PDGFRB* fusions, *FGFR1* neoplasms are usually not sensitive to Imatinib treatment, and specific *FGFR1* inhibitors are currently evaluated in clinical trials [2].

Recently, the MLN‐TK category was expanded to rearrangements of *JAK2*, *FLT3*, *ETV6::ABL1* and others: i.e., *ALK, RET, FGFR2, LYN, NTRK3* in the 5th edition of the WHO (WHO‐WHAEM5) [1] and the ICC [7] showing the growing importance of identifying these specific TK rearrangements.

In most laboratories, the current standard of care (SOC) techniques for the detection of genomic abnormalities in MLN‐TK remain as chromosome banding analysis (CBA) combined with fluorescent in situ hybridization (FISH) and/or reverse transcription‐polymerase chain reaction (RT‐PCR), concentrating on the most frequent aberrations. Today, more extensive testing is recommended, which is not always feasible in a routine setting [2]. In some cases, RNA sequencing (RNA seq) may be performed to identify the fusion partner [6].

Optical genome mapping (OGM) is a relatively novel technology that is rapidly being implemented in cytogenomic laboratories. This methodology is able to detect genome‐wide structural abnormalities (SAs) and copy number aberrations (CNAs) in one single test, by mapping ultra‐long linear DNA molecules, enzymatically labelled at a specific sequence motif occurring about 14–17 times per 100 kb in the human genome [8]. OGM identifies more CNAs as it has a higher resolution than CBA (the latter has a resolution of 5–10 Mb). In contrast to FISH, which is a targeted approach, it provides a whole genome analysis. Even submicroscopic and cryptic SAs can be detected by OGM; as such, it can replace one or more routine diagnostic PCRs. Compared to RNA seq, OGM can detect complex and large structural variants at the genome level, including fusions regardless of expression, but lacks the resolution to confirm fusion transcripts or their functional impact. RNA seq provides high sensitivity and the ability to identify the expression level of the TK fusion but may miss fusions with low expression or degraded RNA.

Furthermore, OGM has the ability to identify new recurrent SAs and CNAs, increasing the diagnostic yield and potentially leading to new treatment options [9].

Here, we evaluate the application of OGM in routine diagnostics of MLN‐TK with known fusions and show its ability to replace the standard FISH panel and some PCR‐based techniques.

## 2 Methods

### 2.1 Sample Selection and Conventional Testing

Ten patients with known or suspected MLN‐TK were selected to assess the ability of OGM to detect recurrent MLN‐TK rearrangements. One case presented clinically as B‐ALL and one as AML. The remaining presented with eosinophilia and/or features of a myeloproliferative disorder. For each sample, SOC tests were performed, i.e., CBA and/or FISH (for *PDGFRB* and *FGFR1*) and/or RT‐PCR for *FIP1L1::PDGFRA* according to standard in‐house procedures [10] or manufacturer's instructions. Additional FISH assays or targeted RNA seq panel testing (Archer FusionPlex Pan‐Heme kit, Integrated DNA Technologies Inc., Iowa, USA) were performed for several cases to identify the fusion partner of *PDGFRB* and to clarify the karyotype or FISH results (Table S1).

Five cases carried recurrent rearrangements in *PDGFRB* (*n* = 2), *FGFR1* (*n* = 1), *JAK2* (*n* = 1) and *SYK* (*n* = 1); three cases had hypereosinophilia but no MLN‐TK fusion according to SOC methods; and two patients presented with cryptic rearrangements (*TNIP1::PDGFRB* and *FIP1L1::PDGFRA*). The *FIP1L1::PDGFRA* fusion was identified in the latter both at diagnosis and relapse.

### 2.2 Optical Genome Mapping

Ten bone marrow (BM) and one peripheral blood (BP) sample were selected for OGM analysis. All samples were stored at −80°C within 6 days of collection. For ethylenediaminetetraacetic acid (EDTA) samples, 650 μL was stored directly at −80°C with or without a 10% mixture of dimethyl sulfoxide (DMSO)‐ fetal calf serum (FCS) (1:1). For heparin samples, 10% 0.5 M EDTA was added before freezing at −80°C with or without a 10% mixture of DMSO:FCS (1:1).

Genomic DNA (gDNA) was extracted as previously described by Rack et al. 2022 [10].

For each sample, we aimed to reach a coverage of at least 300× in order to obtain a theoretical mean variant allele frequency (VAF) sensitivity of 5% (equivalent to aberrations present in heterozygous state in 10% of the cells) [10].

Quality and run parameters were assessed according to the manufacturer's instructions and included the total DNA collected ≥ 150 kb, the map rate (% of Bionano molecules that align to the reference), the N50 (parameter to qualify molecule length) ≥ 20 kb, the N50 ≥ 150 kb, the average label density (in labels/100 kb), the positive and negative label variance (respectively indicating the percentage of the labels absent in the reference and the percentage of the reference labels in the molecules) and the effective coverage of reference [10].

### 2.3 Structural Variant Calling

For the analysis of cancer genomes, the ‘Rare Variant Analysis (RVA) pipeline’ is the preferred choice [8]. If a coverage of 300× is achieved, the RVA detects structural variants at low VAF (5%): translocations > 70 kb, inversions > 70 kb, insertions > 5–50 kb, deletions > 7 kb and duplications > 70 kb [11]. The ‘Copy Number Variation pipeline’ identifies changes in the copy number throughout the entire genome with a VAF down to 10%–15% [8, 11].

For this study the Bionano Access 1.7.2 and Bionano Solve 3.7.2 were used for the analysis of 9/11 samples. The Bionano Access 1.7.1.1 and Bionano Solve 3.7_03302022_283 as well as the Bionano Access 1.8.1 and Bionano Solve 3.8.1 were used in the other cases (Cases 4B and 10, respectively). OGM molecules were mapped to the human reference genome (hg) 38. For data analysis, the filter criteria described in the paper of Levy et al. [8] were used, with additional manual review of the MLN‐TK‐associated genes listed in Table S2.

### 2.4 Comparison of the Results

The OGM results were compared to the results of CBA, and/or FISH and/or RT‐PCR analysis and were considered concordant if the same abnormalities were detected. Variations in assigned breakpoints within the same chromosome arm were not considered discordant. If the abnormalities identified by one of the approaches were inconsistent, the results were considered discordant [10]. Additional molecular testing (RNA seq or other) was performed where necessary to resolve discordant results or to verify the additional information identified by OGM.

## 3 Results

### 3.1 Technical Characteristics of the OGM Analysis

OGM analysis resulted in an average label density of 16/100 kb, a map rate of 82%, and average effective genome coverage of 274×. The quality parameters for each sample included in this study are shown in Table S3.

For 5 samples, the quality parameters were sub‐optimal. Typically, these samples had a lower amount of extracted DNA which resulted in a coverage below the threshold of 300× (Table S3). For Case 5 and 9, we re‐extracted the DNA after adaptation of the white blood cell lysis step, using the Prep SP Blood and cell culture DNA isolation kit‐G1 with modifications (Bionano Genomics, Appendix S1). For both samples, adaptation of the white blood cell lysis step improved the amount of extracted DNA and the coverage (Table S4). Only for sample 9, the N50 ≥ 20/150 kb values were still below the optimal range (Table S4). However, even with suboptimal QA parameters, OGM was able to detect the clinically important rearrangements.

### 3.2 Ability of OGM to Detect Disease Defining Abnormalities

A MLN‐TK disease‐defining TK rearrangement was detected in 7/10 cases by OGM (Table 1). In five of these cases, the TK gene rearrangement had previously been identified by CBA and FISH analysis (Cases 1, 2, 7, 8 and 9).

**TABLE 1:** Overview of the results from CBA, FISH, RT‐PCR and OGM analysis in the study cohort.

	Results		
Sample	Karyotype	FISH/RT‐PCR	OGM
1 [12]	46,XY,t(5;12)(q32;q23)[8]/46,XY[2]	nuc ish (PDGFRBx2)(3'PDGFRB sep 5'PDGFRB)x1[168/200],(FGFR1x2)[196],(D8Z1,JAK2)x2[199] RT‐PCR *FIP1L1::PDGRA*: Neg	ogm[GRCh38] t(5;12)(q32;q23.3)(150126003;108541475)(*SART3::PDGFRB*)
2	46,XY,t(5;14)(q32;q31)[10]	ish t(5;14)(3'PDGFRB+;5'PDGFRB+)[5].nuc ish (PDGFRBx2)(3'PDGFRB sep 5'PDGFRBx1)[165/200] RT‐PCR *FIP1L1::PDGRA*: ND	ogm[GRCh38] t(5;14)(q32;q32.11)(150126003;91315955)(*CCDC88C::PDGFRB*)
3	46,XX[10]	ND RT‐PCR *FIP1L1::PDGRA*: Neg	ogm[GRCh38] (X,1‐22)×2
4A	46,XY[20]	ND RT‐PCR *FIP1L1::PDGRA*: ND but Pos with NGS (Table S1)	ogm[GRCh38] 4q12(53427708_54281141)x1(*FIP1L1::PDGFRA*)
4B	46,XY[10]	ND RT‐PCR *FIP1L1::PDGRA*: ND	ogm[GRCh38] 4q12(53427708_54281141)x1(*FIP1L1::PDGFRA*),11q23.3(118450866_118493942)x3(*KMT2A*‐PTD)
5	46,XY,del(13)(q13q21)[7]/46,XY[3]	nuc ish (PDGFRBx2)[200],(FGFR1x2)[192] RT‐PCR *FIP1L1::PDGRA*: Neg	ogm[GRCh38] 13q12.3q21.2(31368897_60524681)x1
6	46,XY[10]	nuc ish (PDGFRBx2)[198],(FGFR1x2)[195] RT‐PCR *FIP1L1::PDGRA*: Neg	ogm[GRCh38] (X,Y)x1,(1‐22)x2
7	45,X,‐Y,add(8)(p11),der(22)t(8;22)(p11;q11)[9]/45,X,‐Y[1]	ish ?ins(22;8)(q11;p11p?)(BCR+ or BCR++,3'FGFR1+;5'FGFR1+,BCR‐)[10].nuc ish (FGFR1x2)(3'FGFR1 sep 5'FGFR1x1)[188/200],(ABL1x2,BCRx3)[33/200] RT‐PCR *FIP1L1::PDGRA*: ND	ogm[GRCh38] (Y)x0, t(8;8)(p12;p11.23)(29324406;38420475), t(8;22)(p12;q11.23)(29325115;23323104), t(8;22)(p11.23;q11.23)(38408156;23261125)(*BCR ::FGFR1*)
8 [13]	46,XX,t(8;9)(p22;p24)[1]/46,sl,der(8;9)(q10;q10),inc[6]/46,X,t(X;4)(p11;q1?3)[4]/46,XX[11]	ish t(8;9)(p22;p24)(wcp9+,wcp8+,5'PCM1‐,3'JAK2‐; wcp8+,5'PCM1+,3'JAK2+,wcp9+),der(8)t(8;9)(q11;p24)(wcp8+,5'PCM1+,wcp9+,3'JAK2+),der(9)t(8;9)(q11;p11)(wcp8+,5'PCM1‐,wcp9+,3'JAK2‐)[2].nuc ish (5'PCM1,3'JAK2)x2(5'PCM1 con 3'JAK2x1)[60/100] RT‐PCR *FIP1L1::PDGRA*: ND	ogm[GRCh38] t(8;9)(p22;p24.1)(18021361;5060274)(*PCM1::JAK2*),t(8;9)(q12.2;p24.1)(60671418;5961314),9p24.1p22.3(7303095_16259972)x1,9p21.3p21.2(21951394_27261605)×0~1
9 [14]	45,X,‐Y,t(9;12)(q22;p13)[9]/46,XY[1]	nuc ish (RP11‐563G12/RP11‐61N16x2,RP11‐148I22x3)(RP11‐563G12/RP11‐16N16 con RP11‐148I22)x2[107/200],(ETV6)x2[200] RT‐PCR *FIP1L1::PDGRA*: Neg	ogm[GRCh38] (Y)x0,t(9;12)(q22.2;p13.31)(90870096 ;6133766),t(9;12)(q22.2;p13.2)(90870096;11870531),inv(12) )(p13.31p13.2)(6161864_11881907)(*ETV6::SYK*)
10	46,XX[10]	nuc ish (3’PDGFRBx2;5’PDGFRBx1)(3’PDGFRB con 5’PDGFRB)x1[28/100] RT‐PCR *FIP1L1::PDGRA*: Neg	ogm[GRCh38] 5q32q33.1(150126003_151032635)x1~2(*TNIP1::PDGFRB*)

*Note:* Chromosomal locations were obtained from the human December 2013 (GRCH38/hg38) assembly of the UCSC Genome Browser. ISCN 2024.

Abbreviations: CBA: chromosome banding analysis; ish: metaphase FISH; ND: not determined; Neg: negative; nuc ish: interphase FISH; Pos: positive.

**FIGURE 1:**
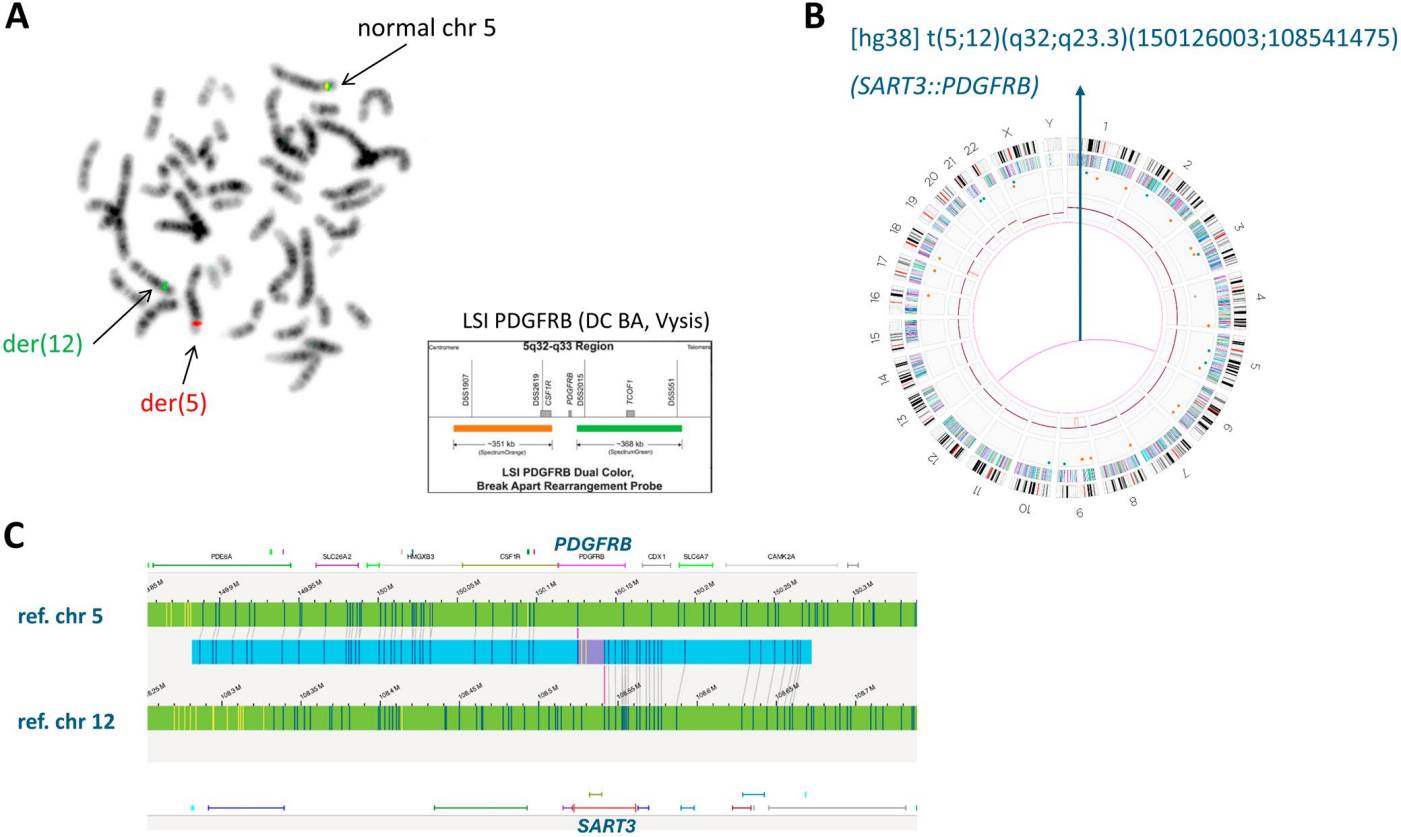
FISH and OGM analysis of Case 1 [12]. (A) Design of the *PDGFRB* BA probe (LSI BA, Vysis) and metaphase showing a split of *PDGFRB* with a 3′ centromeric red signal on der(5) and a 5′ telomeric green signal on der(12), confirming a *PDGFRB*‐rearrangement. (B) The OGM circus plot showed a t(5;12)(q32;q23.3). (C) Detailed analysis (genome browser) of the translocation identified a *SART3::PDGFRB* rearrangement. BA, break apart.

Cases 1 and 2 showed a recurrent *PDGFRB*‐rearrangement by FISH (Figure 1), but the partner genes remained unknown. OGM confirmed the translocations and readily identified their respective fusion partners, *SART3* [12] and *CCDC88C* (Table 1, Figure 1). The *SART3::PDGFRB* fusion was subsequently confirmed by RNA seq as previously described by Van Thillo et al. [12] (Table S1).

**FIGURE 2:**
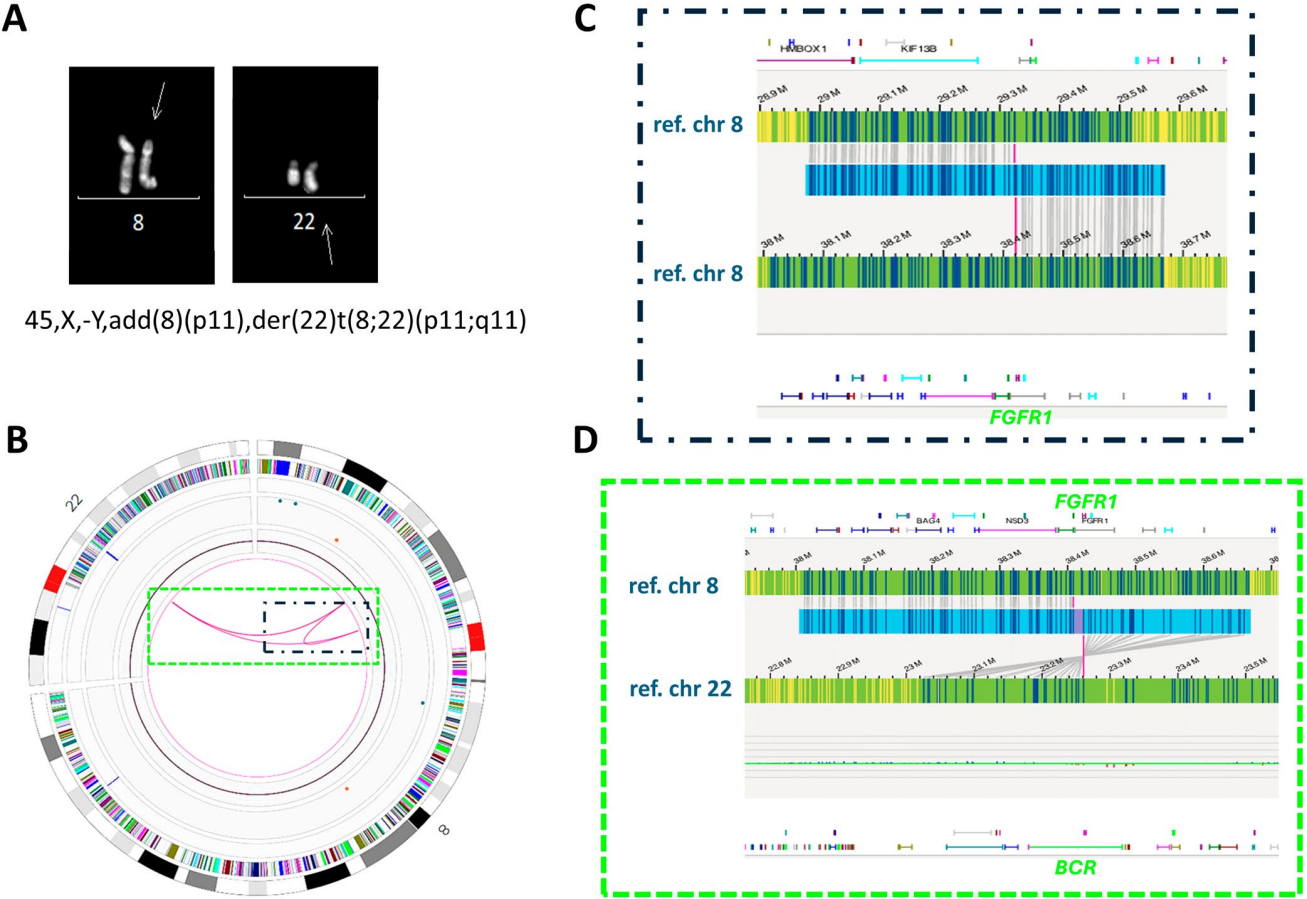
CBA and OGM analysis of Case 7. (A) Representative R‐banded partial metaphase and karyotype of Case 7. (B) The OGM circus plot showed one intrachromosomal and two interchromosomal translocations between chromosome 8 and 22. (C, D) Detailed analysis on the genome browser view showed an inverted insertion of the 8p11.23p12 region into the *BCR* gene on chromosome 22 leading to a *BCR::FGFR1* rearrangement.

For Case 7, CBA/FISH showed a *BCR::FGFR1* fusion associated with loss of the Y chromosome (Figure 2). OGM confirmed these abnormalities and, in addition, elucidated the mechanism leading to the *BCR::FGFR1* rearrangement by demonstrating an inverted insertion of 8p11.23p12 into the *BCR* gene on chromosome 22 (Table 1, Figure 2).

OGM confirmed the translocation t(8;9) observed by CBA in Case 8, revealing the genes involved, *PCM1* and *JAK2*, and showed additional losses on chromosome 9 (Table 1). The *PCM1::JAK2* fusion was confirmed by nested RT‐PCR analysis [13] (Table S1).

Furthermore, in Case 9, CBA identified a t(9;12)(q22;p13). While FISH analysis confirmed the involvement of *SYK*, no rearrangement could be shown for *ETV6*, using a commercial Break Apart probe (LSI *ETV6* DC BA (Vysis, Abbott) [14]. Concerning *SYK*, FISH was performed using homemade dual‐color break apart bacterial artificial chromosomes (BAC) probes RP11‐61N16 and RP11‐563G12 (spectrum green) flanking 5'*SYK* and RP11‐148I22 (spectrum orange) flanking 3'*SYK*. Interestingly, OGM confirmed the t(9;12)(q22.2;p13.2) and could clarify that the *ETV6::SYK* fusion resulted from an inverted insertion of 12p13.31p13.2 into the *SYK* gene on chromosome 9 (Table 1). OGM was also able to correctly locate the breakpoints to exon 5 of *ETV6* and to exon 6 of *SYK* as previously described by Lierman et al. [14] (Table S1).

**FIGURE 3:**
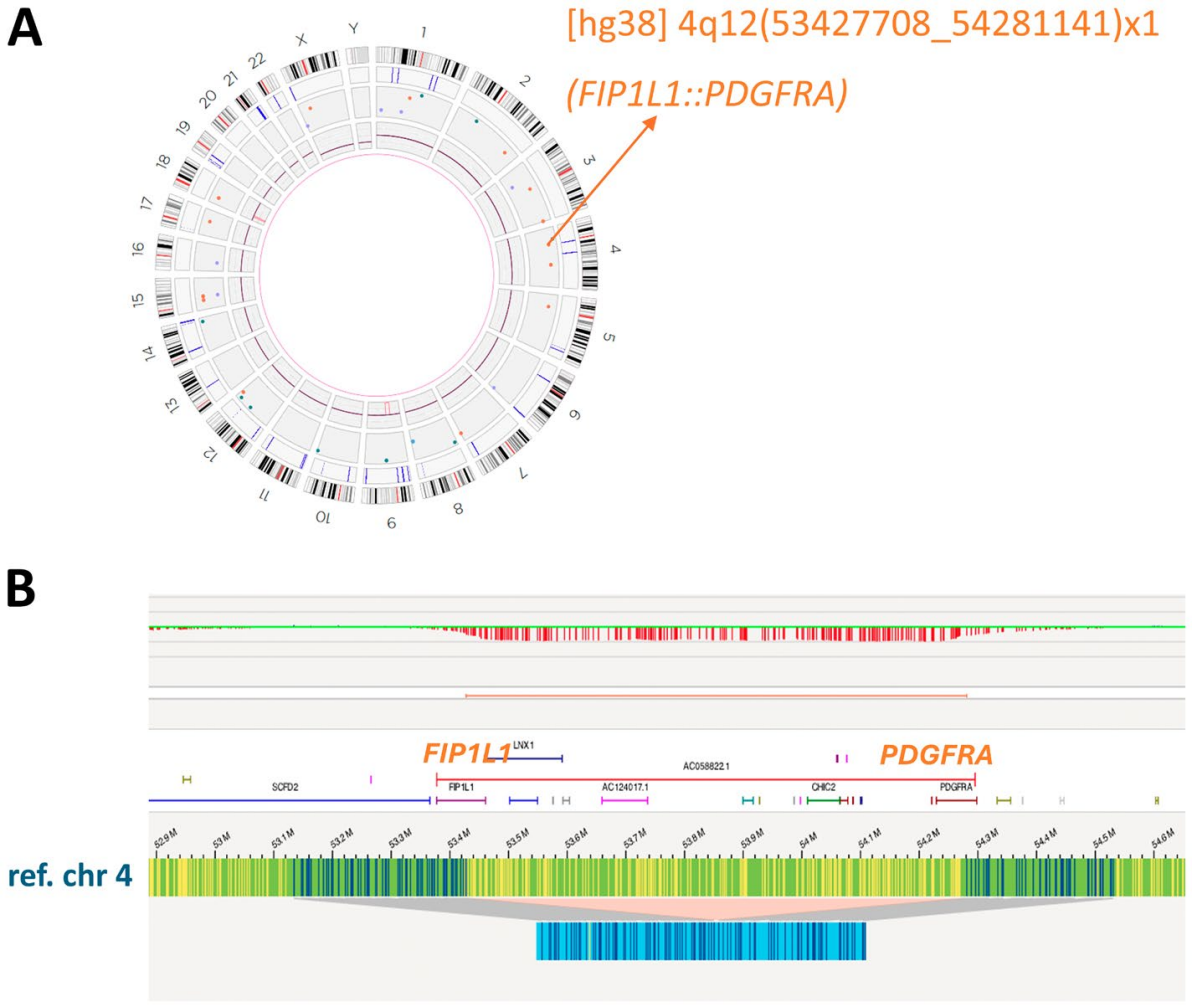
OGM analysis of Case 4A. (A) The circos plot of Case 4A identified a deletion in 4q12. (B) Detailed analysis (genome browser view) of the deletion in 4q12 leading to the *FIP1L1::PDGFRA* fusion.

**FIGURE 4:**
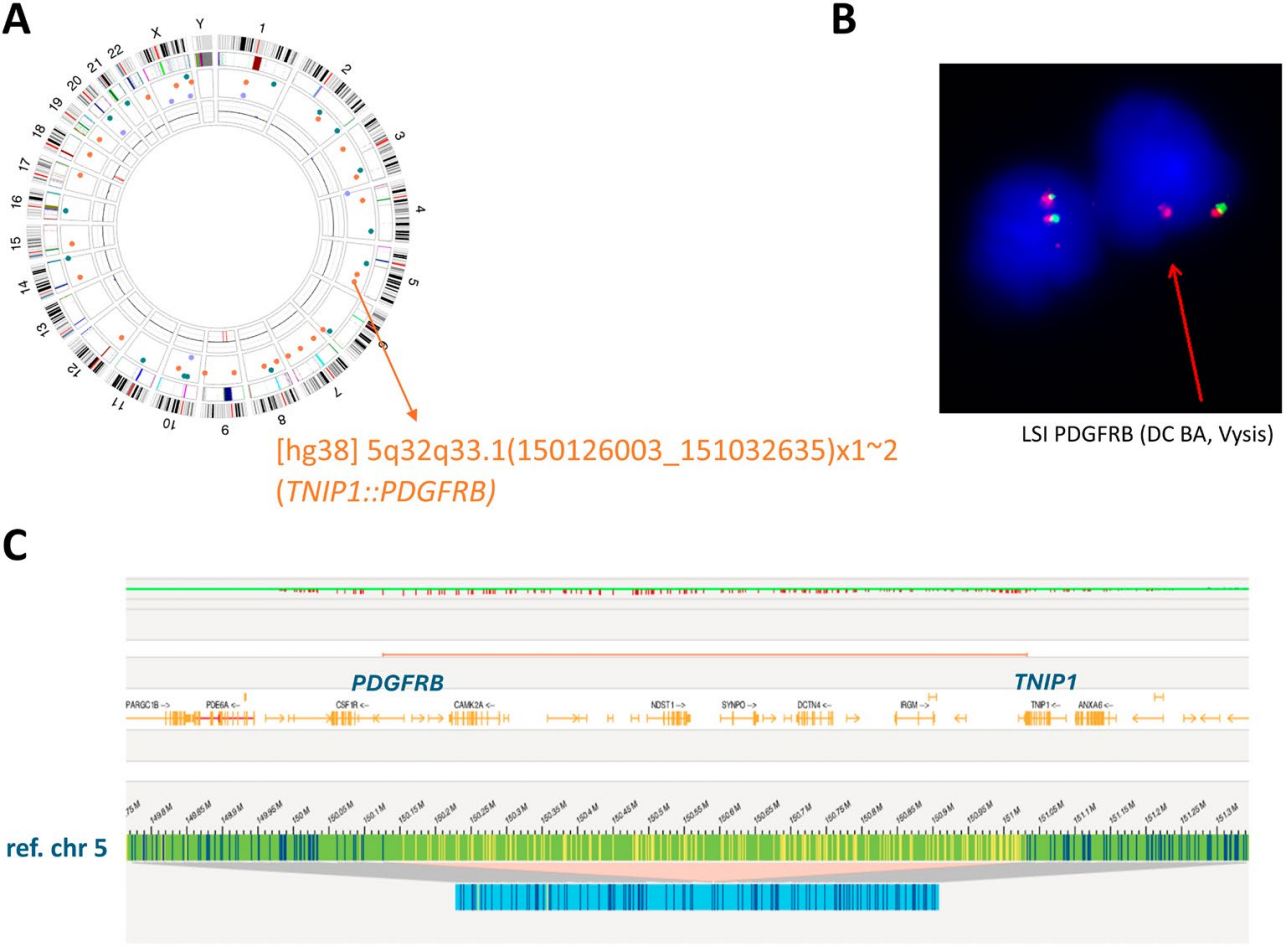
FISH and OGM analysis of Case 10. (A) The circos plot of Case 10 identified a cryptic deletion in 5q32q33.1. (B) Interphase FISH analysis of *PDGFRB* (LSI BA, Vysis, design in Figure 1A) (3′cen (SO)/5′tel. (SG)) detected loss of the 5′ telomeric signal, confirming the deletion in *PDGFRB*. (C) Detailed analysis (genome browser) of the cryptic deletion in 5q32q33.1 leading to the *TNIP1::PDGFRB* fusion. BA, break apart.

In two of the four cases with a normal karyotype, OGM identified a cytogenetically cryptic TK gene rearrangement (Table 1). In Case 4, OGM disclosed a *FIP1L1::PDGFRA* fusion (Figure 3), which was confirmed by NGS analysis (AmpliSeq for Illumina Myeloid Panel) (Table S1). Of note, for this patient, OGM detected the *FIP1L1::PDGFRA* rearrangement both at diagnosis (with a central blast count of 10%, Case 4A) and in peripheral blood at the time of relapse with evolution to secondary AML (with a peripheral blast count of 92%, Case 4B). Similarly, in Case 10, OGM detected a rare cryptic deletion of 5q32q33.1 leading to a *TNIP1::PDGFRB* fusion (Figure 4).

OGM did not detect any aberrations in the two remaining cases with a normal CBA result and confirmed the abnormality detected by CBA (e.g., del(13)(q13q21)) in a further case, but did not identify any additional TK aberration (respectively Cases 3, 6 and 5).

### 3.3 Analysis of Additional Clonal Abnormalities Detected by SOC Techniques Versus OGM

Using CBA, six of the 10 cases presented clonal abnormalities. For two patients, the MLN‐TK rearrangement was present as the sole abnormality (Cases 1 and 2), while 4 others had other or additional abnormalities in the karyotype (Cases 5, 7, 8 and 9). OGM results were concordant with the karyotype to detect these other or additional abnormalities, which were identified in the MLN‐TK pathogenic clones (Cases 7, 8 and 9). Only for Case 8, OGM was unable to acknowledge the presence of another independent clone.

Interestingly, OGM detected a *KMT2A* partial tandem duplication (*KMT2A*‐PTD), which was not present in the diagnostic sample (Case 4A) at the time of secondary AML development (Case 4B).

## 4 Discussion

In this study, we evaluated the use of OGM in the routine diagnostic workflow of suspected MLN‐TK as compared to our standard testing pathway. Overall, there was a 100% concordance between the conventional approach and OGM in this series, with all cases having concordant OGM results compared to CBA, FISH and/or RT‐PCR analysis for the MLN‐TK fusions.

Additionally, OGM enhanced the diagnostic yield in the current cohort by detecting the cytogenetically cryptic *TNIP1::PDGFRB* fusion. This rare fusion had previously been reported by two groups in both an adult and a paediatric patient [15, 16]. Also, OGM identified a cryptic *ETV6::SYK* fusion resulting from an inverted insertion of 12p13.31p13.2. Although an *ETV6* rearrangement was suspected given the presence of a translocation with a 12p13 breakpoint, no rearrangement could be identified by FISH analysis [14].

Moreover, OGM identified the fusion partner in two *PDGFRB*‐rearranged cases and one *JAK2*‐rearranged case reported by CBA/FISH. In the latter, OGM showed an additional translocation t(8;9)(q12.2;p24.1) and loss of 9p, besides the t(8;9)(p22;p24) which gave rise to the *PCM1::JAK2* fusion [13]. Correct identification of the partner genes is important as they play a role in disease classification and could impact response to treatment [17, 18].

Also, OGM elucidated the mechanism leading to a *BCR::FGFR1* rearrangement by demonstrating an inverted insertion of the 8p11.23p12 region into the *BCR* gene on chromosome 22. Similarly, research by Vanjari et al. showed that OGM provided additional insights into the chromosomal mechanism leading to a *BCR::JAK2* rearrangement in a case with a myeloproliferative neoplasm, not otherwise specified [19].

Several studies compared OGM with conventional cytogenetic testing in different haematological malignancies such as AML [20, 21], ALL [22, 23], MDS [24] and chronic lymphocytic leukaemia (CLL) [25, 26]. All of these demonstrated that OGM has equal or better sensitivity and resolution for the detection of diagnostic and prognostic abnormalities, with only a few discordances [8]. Likewise, in this study, OGM did not detect the t(X;4)(p11;q1?3) in Case 8, probably because of the location of the breakpoint close to the centromeric region of chromosome X, which contains a lot of repetitive sequences [8, 10].

The use of OGM for the detection of fusion genes in myeloid HES (hypereosinophilic syndromes) was previously reported by Podvin et al., who analysed two cases presenting 5q31 rearrangements in the karyotype [27]. Additional cryptic SAs and CNAs were detected in both cases [27]. In our analysis, OGM also identified extra abnormalities not reported by SOC methods, such as the *KMT2A*‐PTD.

Of note, we encountered technical challenges with DNA extraction for certain samples in our cohort. Five samples initially had sub‐optimal quality parameters presenting low levels of total DNA ≥ 150 kb (Gbp) and an effective coverage below 300×. We found that by using proteinase K, Lysis and Binding Buffer (LBB) from the Prep SP Blood and cell culture DNA isolation kit‐G1 with modifications and phenylmethylsulfonyl fluoride (PMSF) for the white blood cell lysis step, the DNA quality could significantly be improved. Still, further research and optimisation on additional samples are needed to fully refine this methodology.

Indeed, a limitation of this single center study is the small and non‐consecutive cohort. Larger (multicenter) studies will be required to further evaluate the full potential of OGM in the diagnostic workflow.

Here, we demonstrated that OGM can replace FISH, CBA and/or PCR‐based techniques or RNA seq in routine genetic diagnostics of patients with suspicion of MLN‐TK. Moreover, OGM has the potential to detect new recurrent structural aberrations and to identify previously unknown fusion partners, which will enable individualised patient treatment and improve follow‐up analysis. Still, the prior exclusion of *FIP1L1::PDGFRA* positive cases by RT‐PCR might be advised to reduce costs and to reserve the analysis for patients with persisting, unexplained eosinophilia and related organ damage.

## Author Contributions

**Justine Vanhevel:** conceptualization (equal), data curation (equal), formal analysis (equal), validation (equal), visualization (equal), writing – original draft (equal). **Katrina Rack:** conceptualization (equal), data curation (equal), formal analysis (equal), writing – original draft (equal). **Geneviève Ameye:** conceptualization (equal), data curation (equal), formal analysis (equal), writing – review and editing (equal). **Hayat Mokrani:** methodology (equal), writing – review and editing (equal). **Jolien De Bie:** funding acquisition (equal), investigation (equal), validation (equal), writing – review and editing (equal). **Lucienne Michaux:** conceptualization (equal), funding acquisition (equal), investigation (equal), validation (equal), writing – review and editing (equal). **Barbara Dewaele:** conceptualization (equal), data curation (equal), formal analysis (equal), funding acquisition (equal), methodology (equal), software (equal), validation (equal), writing – original draft (equal).

## Ethics Statement

The study was conducted in compliance with the principles of the Declaration of Helsinki, Good Clinical Practice and all applicable regulatory requirements. Approval by the Ethical Committee of the University Hospitals Leuven (Belgium) was obtained.

## Conflicts of Interest

The authors declare no conflicts of interest.

## Supporting information


Appendix S1.

**Table S1.** Confirmation of the TK gene rearrangements found by OGM.

**Table S2.** Important genomic regions in MLN‐TK.

**Table S3.** OGM quality parameters of the 11 MLN‐TK samples analysed in the current study.

**Table S4.** OGM quality parameters of the 2 MLN‐TK samples after adaptation of the protocol to improve quality parameters.

## Data Availability

Original data and protocols are available upon specific request.
