# Supplementary material for: The Use of Optical Genome Mapping for the Detection of Tyrosine Kinase Gene Fusions in Myeloid/Lymphoid Neoplasms
Source: J Cell Mol Med. 2025 Jun 18;29(12):e70640. doi: 10.1111/jcmm.70640 (PMC12176696; doi:10.1111/jcmm.70640)
Supplement: Supplementary file 1 — Appendix S1. [file JCMM-29-e70640-s002.docx]

# Supplementary Methods

***Adaptation to the protocol to improve quality parameters for MLN-TK samples (bone marrow)***

After washing the bone marrow samples and counting the number of white blood cells (WBC), 1,5.10^6^ cells were resuspended in cold stabilizing buffer and placed on ice. To lyse the WBC, 50 µl of proteinase K and 20 µl of RNAse A (Prep SP Blood and cell culture DNA isolation kit-G1 with modifications) were added to the cells. Samples were mixed by pipetting slowly. After 3 minutes (min) of incubation at room temperature (RT), 225 µl of LBB buffer (with detergent inserted, Prep SP Blood and cell culture DNA isolation kit-G1 with modifications) was added to the samples and then the buffered samples were homogenized by inverting 15 times. Subsequently, samples were incubated on the Hulamixer for 15 min at 10 RPM and centrifuged for 2 seconds (sec) before addition of 10 µl PMSF (100 mM) (Sigma-Aldrich). After homogenization by inverting the samples 5 times, samples were centrifugated 2 sec and incubated at RT for 10 min, after which the Nanodisks (Prep SP Blood and cell culture DNA isolation kit-G1 with modifications) were added. Next, 340 µl 100% isopropanol (Sigma-Aldrich) was added to the samples. After homogenization by inverting the samples 5 times, the manufacturer’s protocol from the SP Blood & cell culture DNA Isolation kit-G2 (Bionano Genomics) was continued with incubation on the Hulamixer for 15 min at 10 RPM. All products (proteinase K, LBB buffer and Nanodisks) are also available in the Prep SP Tissue and Tumor DNA isolation Kit (Bionano Genomics).
