# Supplementary material for: The Use of Optical Genome Mapping for the Detection of Tyrosine Kinase Gene Fusions in Myeloid/Lymphoid Neoplasms
Source: J Cell Mol Med. 2025 Jun 18;29(12):e70640. doi: 10.1111/jcmm.70640 (PMC12176696; doi:10.1111/jcmm.70640)
Supplement: Supplementary file 2 — Table S1. Confirmation of the TK gene rearrangements found by OGM. [file JCMM-29-e70640-s004.docx]

***Supplementary Table S1****: Confirmation of the TK gene rearrangements found by OGM.*

|  | **Results** | | |
| --- | --- | --- | --- |
| **sample** | **Rearrangement detected by OGM** | **Fusion partner** | **Confirmed by** |
| 1 | *SART3::PDGFRB* | *SART3* | **RNA seq:**  *SART3* [NM_014706.4] exon 15::*PDGFRB* [NM_002609.4] exon 12 ^12^ |
| 4A | *FIP1L1::PDGFRA* | *FIP1L1* | **NGS** (AmpliSeq for Illumina Myeloid Panel) (data not shown, analysis done in different center) |
| 7 | *BCR::FGFR1* | *BCR* | **FISH:**  ish ?ins(22;8)(q11;p11p?)(BCR+ or BCR++,3'FGFR1+;BCR-,5'FGFR1+)[10].nuc ish (FGFR1)x2(3'FGFR1 sep 5'FGFR1)x1[188/200],(ABL1x2,BCRx3)[33/200] |
| 8 | *PCM1::JAK2* | *PCM1* | **Nested RT-PCR:**  *PCM1* exon 36:: *JAK2* exon 9 ^13^ |
| 9 | *ETV6::SYK* | *ETV6* | **Targeted Locus Amplification (TLA-technology):**  *ETV6* exon 5 :: *SYK* exon 6 ^14^ |
