# Supplementary material for: The Use of Optical Genome Mapping for the Detection of Tyrosine Kinase Gene Fusions in Myeloid/Lymphoid Neoplasms
Source: J Cell Mol Med. 2025 Jun 18;29(12):e70640. doi: 10.1111/jcmm.70640 (PMC12176696; doi:10.1111/jcmm.70640)
Supplement: Supplementary file 3 — Table S2. Important genomic regions in MLN‐TK. [file JCMM-29-e70640-s003.docx]

***Supplementary Table S2****: important genomic regions in MLN-TK.*

| **Gene involved** | **Region (hg38)** | **Region (hg19)** |
| --- | --- | --- |
| *PDGFRA* | Chr4: 54,229,280-54,298,245 | Chr4: 55,095,447-55,164,412 |
| *PDGFRB* | Chr5: 150,113,839-150,155,872 | Chr5: 149,493,402-149,535,435 |
| *FGFR1* | Chr8: 38,400,215-38,468,834 | Chr8: 38,257,733-38,326,352 |
| *JAK2* | Chr9: 4,984,390-5,129,948 | Chr9: 4,984,390-5,129,948 |
| *FLT3* | Chr13: 28,003,274-28,100,592 | Chr13: 28,577,411-28,674,729 |
| *SYK* | Chr9: 90,801,787-90,898,549 | Chr9: 93,564,069-93,660,831 |
