# Supplementary material for: The Use of Optical Genome Mapping for the Detection of Tyrosine Kinase Gene Fusions in Myeloid/Lymphoid Neoplasms
Source: J Cell Mol Med. 2025 Jun 18;29(12):e70640. doi: 10.1111/jcmm.70640 (PMC12176696; doi:10.1111/jcmm.70640)
Supplement: Supplementary file 4 — Table S3. OGM quality parameters of the 11 MLN‐TK samples analysed in the current study. [file JCMM-29-e70640-s001.docx]

***Supplementary Table S3****: OGM quality parameters of the 11 MLN-TK samples analyzed in the current study.*

| **Quality parameters** | **N50 ≥ 20 kb (kb)** | **N50 ≥ 150 kb (kb)** | **Total DNA ≥ 150 kb (Gbp)** | **Average label density/ 100 kb** | **Map rate (%)** | **Effective coverage (x)** | **PLV (%)** | **NLV (%)** |
| --- | --- | --- | --- | --- | --- | --- | --- | --- |
| Optimal values | >150 | >230 | >1300 | 14-17 | 70-90 | >300x | 3-6 | 9-15 |
|  |  |  |  |  |  |  |  |  |
| **Sample** |  |  |  |  |  |  |  |  |
| 1 | 169.13 | 284.00 | 1033.01 | 16.37 | 67.8 | 179.10 | 3.50 | 13.80 |
| 2 | 108.75 | 211.13 | 1505.32 | 14.73 | 78.5 | 318.70 | 2.24 | 13.50 |
| 3 | 220.88 | 278.22 | 1531.50 | 14.14 | 83.5 | 366.19 | 5.11 | 13.16 |
| 5 | 349.88 | 349.88 | 479.86 | 14.95 | 94.7 | 127.09 | 3.14 | 8.80 |
| 6 | 258.75 | 319.50 | 1417.10 | 15.47 | 92.7 | 365.42 | 4.49 | 8.48 |
| 7 | 223.50 | 275.63 | 1464.04 | 15.51 | 91.7 | 384.49 | 3.01 | 9.64 |
| 8 | 129.75 | 206.25 | 776.25 | 14.24 | 75.9 | 165.47 | 3.11 | 12.99 |
| 9 | 133.88 | 282.38 | 1045.47 | 15.10 | 81.0 | 228.42 | 4.66 | 12.34 |
| ***Samples with additional aberrations detected by Bionano OGM*** | | | | | | | | |
| 4A | 182.63 | 252.38 | 1535.51 | 14.16 | 88.3 | 391.36 | 1.89 | 13.51 |
| 4B | 208.88 | 282.38 | 1404.00 | 17.37 | 80.7 | 307.17 | 2.39 | 9.41 |
| 10 | 145.50 | 359.13 | 1082.45 | 19.33 | 62.0 | 185.51 | 3.11 | 14.37 |

*Values outside the optimal range are indicated in red (10% of tolerance) ^8^. N50: parameter to qualify the molecule length (≥20 kb and ≥150 kb); PLV: positive labeling variance; NLV: negative labeling variance ^10^.*
