# Supplementary material for: The Use of Optical Genome Mapping for the Detection of Tyrosine Kinase Gene Fusions in Myeloid/Lymphoid Neoplasms
Source: J Cell Mol Med. 2025 Jun 18;29(12):e70640. doi: 10.1111/jcmm.70640 (PMC12176696; doi:10.1111/jcmm.70640)
Supplement: Supplementary file 5 — Table S4. OGM quality parameters of the 2 MLN‐TK samples after adaptation of the protocol to improve quality parameters. [file JCMM-29-e70640-s005.docx]

***Supplementary Table S4****: OGM quality parameters of the 2 MLN-TK samples after adaptation of the protocol to improve quality parameters.*

| **Quality parameters** | **N50 ≥ 20 kb (kb)** | **N50 ≥ 150 kb (kb)** | **Total DNA ≥ 150 kb (Gbp)** | **Average label density/ 100 kb** | **Map rate (%)** | **Effective coverage (x)** | **PLV (%)** | **NLV (%)** |
| --- | --- | --- | --- | --- | --- | --- | --- | --- |
| Optimal values | >150 | >230 | >1300 | 14-17 | 70-90 | >300x | 3-6 | 9-15 |
|  |  |  |  |  |  |  |  |  |
| **Sample** |  |  |  |  |  |  |  |  |
| 5 (before) | 349.88 | 349.88 | 479.86 | 14.95 | 94.7 | 127.09 | 3.14 | 8.80 |
| 5 (after) | 151.13 | 227.25 | 1512.03 | 15.19 | 87.2 | 371.21 | 4.41 | 8.70 |
| 9 (before) | 133.88 | 282.38 | 1045.47 | 15.10 | 81.0 | 228.42 | 4.66 | 12.34 |
| 9 (after) | 117.75 | 195.75 | 1525.79 | 15.33 | 76.7 | 323.90 | 3.72 | 9.06 |

*Values outside the optimal range are indicated in red (10% of tolerance) ^8^. N50: parameter to qualify the molecule length (≥20 kb and ≥150 kb); PLV: positive labeling variance; NLV: negative labeling variance ^10^.*
